# Serratia marcescens DUF1471-Containing Protein SrfN Is Needed for Adaptation to Acid and Oxidative Stresses

**DOI:** 10.1128/msphere.00212-22

**Published:** 2022-10-11

**Authors:** Anna A. Elistratova, Lilia E. Matrosova, Irina V. Khilyas, Tatiana V. Shirshikova, Iuliia V. Danilova, Alexander V. Laikov, Yulia D. Romanova, Cecilia G. Sierra-Bakhshi, Margarita R. Sharipova, Lydia M. Bogomolnaya

**Affiliations:** a Department of Microbiology, Institute of Fundamental Medicine and Biology, Kazan (Volga Region) Federal University, Kazan, Russia; b OpenLab “Omics Technologies,” Institute of Fundamental Medicine and Biology, Kazan (Volga Region) Federal University, Kazan, Russia; c Medical Research Center Immunculus, Moscow, Russia; d Department of Biomedical Sciences, Joan C. Edwards School of Medicine, Marshall University, Huntington, West Virginia, USA; University of Iowa

**Keywords:** DUF1471-containing protein, *Serratia marcescens*, SrfN, oxidative stress

## Abstract

Bacteria can quickly adapt to constantly changing environments through a number of mechanisms, including secretion of secondary metabolites, peptides, and proteins. Serratia marcescens, an emerging pathogen with growing clinical importance due to its intrinsic resistance to several classes of antibiotics, can cause an array of infections in immunocompromised individuals. To better control the spread of S. marcescens infections, it is critical to identify additional targets for bacterial growth inhibition. We found that extracellular metabolites produced by the wild-type organism in response to peroxide exposure had a protective effect on an otherwise-H_2_O_2_-sensitive *ΔmacAB* indicator strain. Detailed analysis of the conditioned medium demonstrated that the protective effect was associated with a low-molecular-weight heat-sensitive and proteinase K-sensitive metabolite. Furthermore, liquid chromatography-tandem mass spectrometry analysis of the low-molecular-weight proteins present in the conditioned medium led to identification of the previously uncharacterized DUF1471-containing protein TBU67220 (SrfN). We found that loss of the *srfN* gene did not have an impact on the production of extracellular enzymes. However, the S. marcescens mutant lacking SrfN was significantly more sensitive to growth in medium with a low pH and to exposure to oxidative stress. Both defects were fully rescued by complementation. Thus, our results indicate that SrfN, a low-molecular-weight DUF1471-containing protein, is involved in S. marcescens SM6 adaptation to adverse environmental conditions.

**IMPORTANCE** Serratia marcescens is ubiquitous in the environment and can survive in water, soil, plants, insects, and animals, and it can also cause infections in humans. In the face of disturbances such as oxidative or low-pH stress, bacteria adapt, survive, and recover through several mechanisms, including changes in their secretome. We show that a hydrogen peroxide-exposed S. marcescens milieu contains a small previously uncharacterized DUF1471-containing protein similar to the SrfN protein in Salmonella enterica serovar Typhimurium, and we illustrate the role of this protein in bacterial survival during acid and oxidative stresses.

## INTRODUCTION

Bacteria can successfully exist in constantly changing environments due to their ability to quickly adapt to new ecological niches. Serratia marcescens, a Gram-negative bacterium from the order *Enterobacterales*, can thrive in a diverse number of habitats ranging from soil, water, plants, the digestive tracts of various animals, to hospital surfaces. The ubiquity of *Serratia* is in part attributed to the variety of compounds that it releases into the environment. Specifically, S. marcescens is known to secrete secondary metabolites as well as a number of potent lytic enzymes, including but not limited to lipase, nuclease, and proteases. These enzymes play important roles in access to nutrients and destruction of competitors (1–6).

A family of low-molecular-weight secreted proteins with a conserved domain of unknown function, DUF1471, was discovered over 20 years ago (7). However, the small size of these proteins, with less than 100 amino acids in their composition, makes their detection and the follow-up analysis more challenging compared to that for larger proteins. Several studies have focused on the identification of the role/s these proteins may play in the biological processes of bacteria. Currently, the DUF1471 family includes several hundred known or predicted proteins, all of which are found in bacteria from the order *Enterobacterales*. Many of these bacterial species contain several genes encoding paralogs of the DUF1471 domain-containing proteins in their genome. To better understand the function of DUF1471-containing proteins, three representative Salmonella enterica serovar Typhimurium proteins (SrfN, YahO, and SssB) were selected by the Northeast Structural Genomics Consortium for detailed structural characterization. This analysis showed that the first 21 amino acid residues from the N terminus of each of these proteins are cleaved during export to the periplasm via a Sec-dependent mechanism. All analyzed proteins had a common fold and contained several beta-sheets and alpha-helices (8). Proteins with the DUF1471 domain are often synthesized under stress conditions (oxidative stress or stress or caused by a decrease of pH in the nutrient medium), as well as during colonization of various surfaces (including the formation of biofilms) (9–15). The exact role(s) the DUF1471-containing proteins play in bacterial survival under stress conditions is unclear, but it was proposed to be related to a change in the characteristics of the cell surface (8).

Here, we show that the wild-type Serratia marcescens SM6 secretes a low-molecular-weight heat-sensitive and proteinase K-sensitive metabolite(s) that is sufficient to rescue the H_2_O_2_-mediated killing of the *ΔmacAB* indicator strain exposed to peroxide. We also show that a small hypothetical protein, TBU67220, is present in medium preconditioned by growth of wild-type S. marcescens. We propose to name this protein SrfN, based on its amino acid homology, predicted structure similarity, and the evolutionary conservation to the previously characterized DUF1471-containing SrfN protein in Salmonella Typhimurium. We show that S. marcescens SrfN does not have an impact on the production of extracellular enzymes. Nevertheless, we show that the mutant strain lacking *srfN* is more sensitive to exposure to the low-pH conditions and to peroxide-containing medium required for S. marcescens fitness during acid and oxidative stresses. Both defects were fully restored by complementation. We conclude that the DUF1471-containing protein SrfN, identified in the medium preconditioned by growth of the peroxide-exposed wild-type strain, is required for S. marcescens fitness during acid and oxidative stresses.

## RESULTS

### Serratia marcescens secretes metabolites needed for bacterial growth in the presence of hydrogen peroxide.

**FIG 1:**
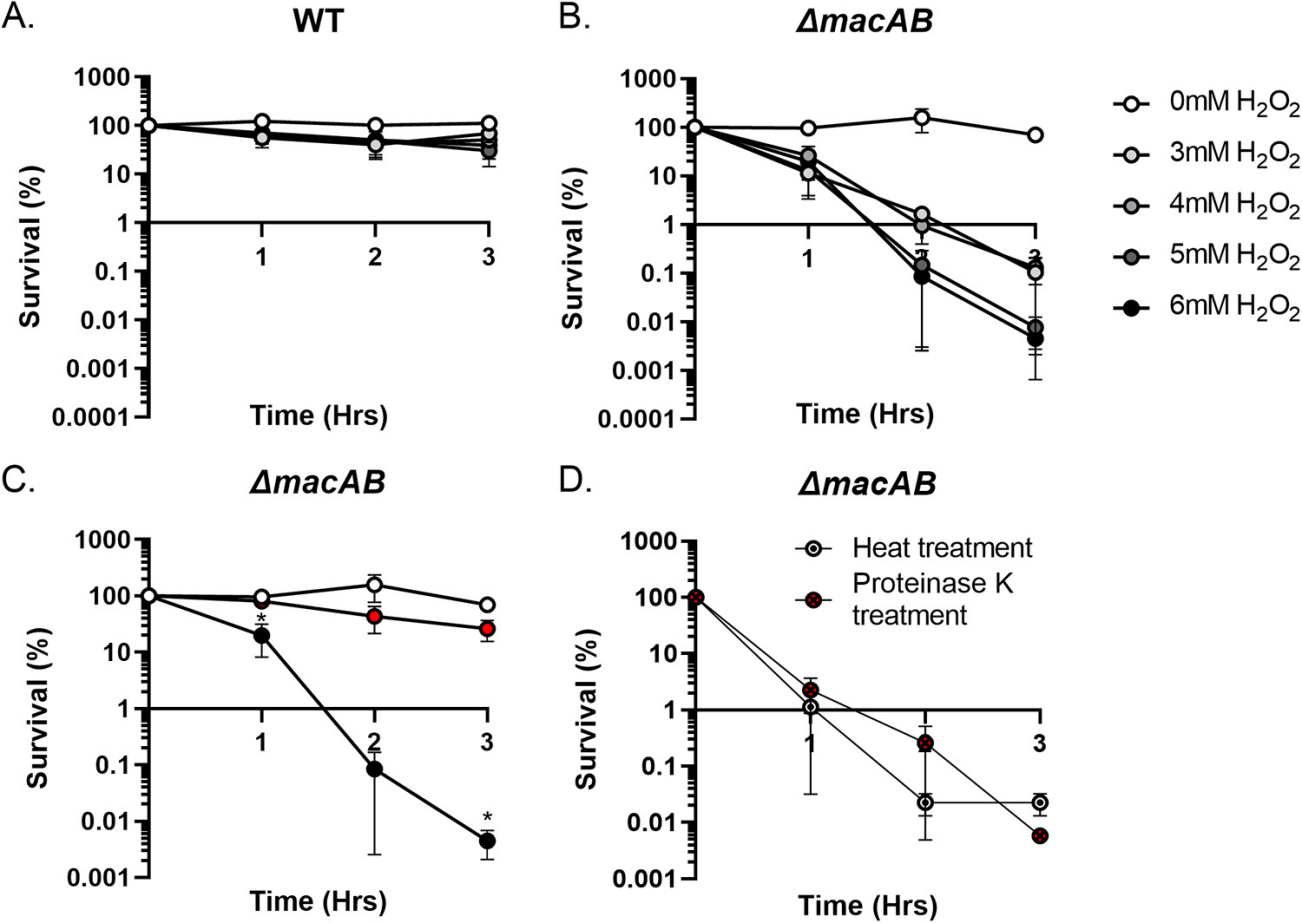
Metabolites present in the minimal medium preconditioned by S. marcescens wild type supported growth of the peroxide-sensitive *ΔmacAB* mutant strain in the presence of H_2_O_2_. (A and B) Overnight cultures of the wild type (A) and the peroxide-sensitive *ΔmacAB* mutant strain (B) were grown in LB broth, washed, and used for inoculation of glycerol-containing minimal M9 broth containing 0, 3, 4, 5, or 6 mM H_2_O_2_. Aliquots were collected hourly, serially diluted, and plated. (C) An overnight culture of the *ΔmacAB* mutant strain was grown in LB broth, washed, and used for inoculation of M9 broth containing 0 mM (white circles) or 6 mM (black circles) hydrogen peroxide. In addition, the preconditioned M9 medium was prepared by the growth of wild-type S. marcescens in the presence of 6 mM H_2_O_2_ for 4 h, followed by centrifugal supernatant separation with subsequent filtration using a 0.2-μm filter. The collected preconditioned medium was supplemented with 6 mM H_2_O_2_ and used for growth of the *ΔmacAB* mutant strain (red circles). (D) The preconditioned medium from panel C was either boiled for 10 min (circle with a dot) or treated with proteinase K (red circle with an X). After each treatment, medium was supplemented with 6 mM H_2_O_2_ and used for growth of the *ΔmacAB* mutant strain. Aliquots were collected hourly, serially diluted, and plated. Data represent the survival means from three independent experiments and standard errors. *, *P* < 0.05 (unpaired *t* test).

Bacteria use a number of strategies to survive adverse environmental conditions. Recent studies in Salmonella have shown that bacterial survival during oxidative stress requires not only the presence of anti-H_2_O_2_ enzymes (catalases and peroxidases) (16) but also secretion of low-molecular-weight metabolites (17, 18). To determine whether secreted metabolites are also needed for growth of Serratia marcescens, another bacterium from the order *Enterobacterales*, we first evaluated the growth of wild type and the peroxide-sensitive *ΔmacAB* mutant strain in minimal medium M9 containing various amounts of hydrogen peroxide. The mutant lacking the MacAB efflux pump cannot survive peroxide exposure in rich LB broth (19). Similar to these previous experiments performed with LB broth (19), wild-type S. marcescens remained viable after 3 h of growth in minimal medium containing 6 mM H_2_O_2_ (Fig. 1A). In stark contrast, approximately 80% of bacteria in the *ΔmacAB* mutant culture were killed within 1 h of growth in the medium containing 3 mM peroxide. Furthermore, the viability of this mutant strain was completely lost after 3 h of growth in the presence of 6 mM H_2_O_2_ (Fig. 1B).

Next, we obtained a cell-free minimal medium preconditioned by 4-h cultivation of wild type bacteria in the presence of peroxide supplemented with fresh 6 mM H_2_O_2_, and we used this resulting broth to test growth of the *ΔmacAB* mutant. We found that the putative metabolites present in the preconditioned medium were able to support growth of the *ΔmacAB* indicator strain (Fig. 1C). Interestingly, both heat and proteinase K treatments completely abolished the ability of preconditioned medium to rescue growth of the *ΔmacAB* mutant strain (Fig. 1D). Thus, we concluded that wild-type S. marcescens secretes a heat- and proteinase K-sensitive metabolite(s) that helps bacteria survive the oxidative stress.

### The metabolite(s) supporting growth of the indicator *ΔmacAB* strain during oxidative stress has a low molecular weight.

**FIG 2:**
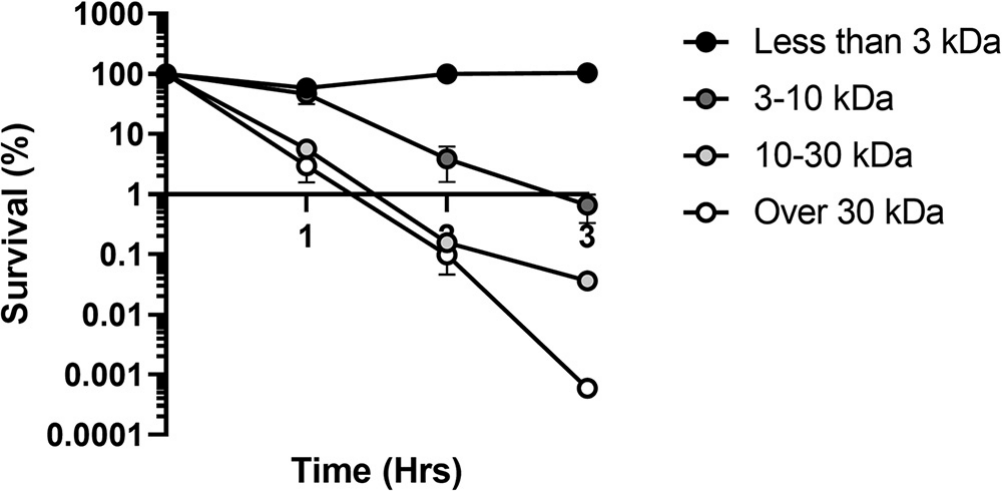
Growth of the indicator *ΔmacAB* strain in peroxide-containing medium was supported by low-molecular-weight (<10-kDa) metabolites. Preconditioned M9 medium, prepared as described for Fig. 1, was separated into four fractions by using Amicon Ultra centrifugal filter units with a 3-, 10-, or 30-kDa molecular weight cutoff. Each fraction was supplemented with 6 mM H_2_O_2_ and used for growth of the *ΔmacAB* mutant strain. Aliquots were collected hourly, serially diluted, and plated. Data represent the survival means from three independent experiments and the standard errors.

Because the conditioned medium supported growth of an otherwise-peroxide-sensitive *ΔmacAB* indicator strain, we next investigated whether this protection was associated with the presence of molecules of a particular size. Using centrifugal filters with different molecular weight cutoffs, we separated conditioned medium into four fractions based on the molecular sizes of metabolites: fraction 1 (metabolites with a molecular weight of <3 kDa); fraction 2 (metabolites with a molecular weight between 3 and 10 kDa); fraction 3 (metabolites with a molecular weight between 10 and 30 kDa); fraction 4 (metabolites with a molecular weight of >30 kDa). Each fraction was tested for its ability to rescue peroxide sensitivity of the *ΔmacAB* indicator strain. We found that fractions containing metabolites with molecular sizes less than 3 kDa (fraction 1) and between 3 and 10 kDa (fraction 2) resulted in a complete and a partial restoration, respectively, of the *ΔmacAB* mutant growth in the presence of peroxide (Fig. 2). These results indicated that the metabolite(s) needed for the growth of the peroxide-sensitive S. marcescens strain has a relatively low molecular weight (<10 kDa).

### DUF1471-containing protein is present in the low-molecular-weight fraction of the precondition medium.

**FIG 3:**
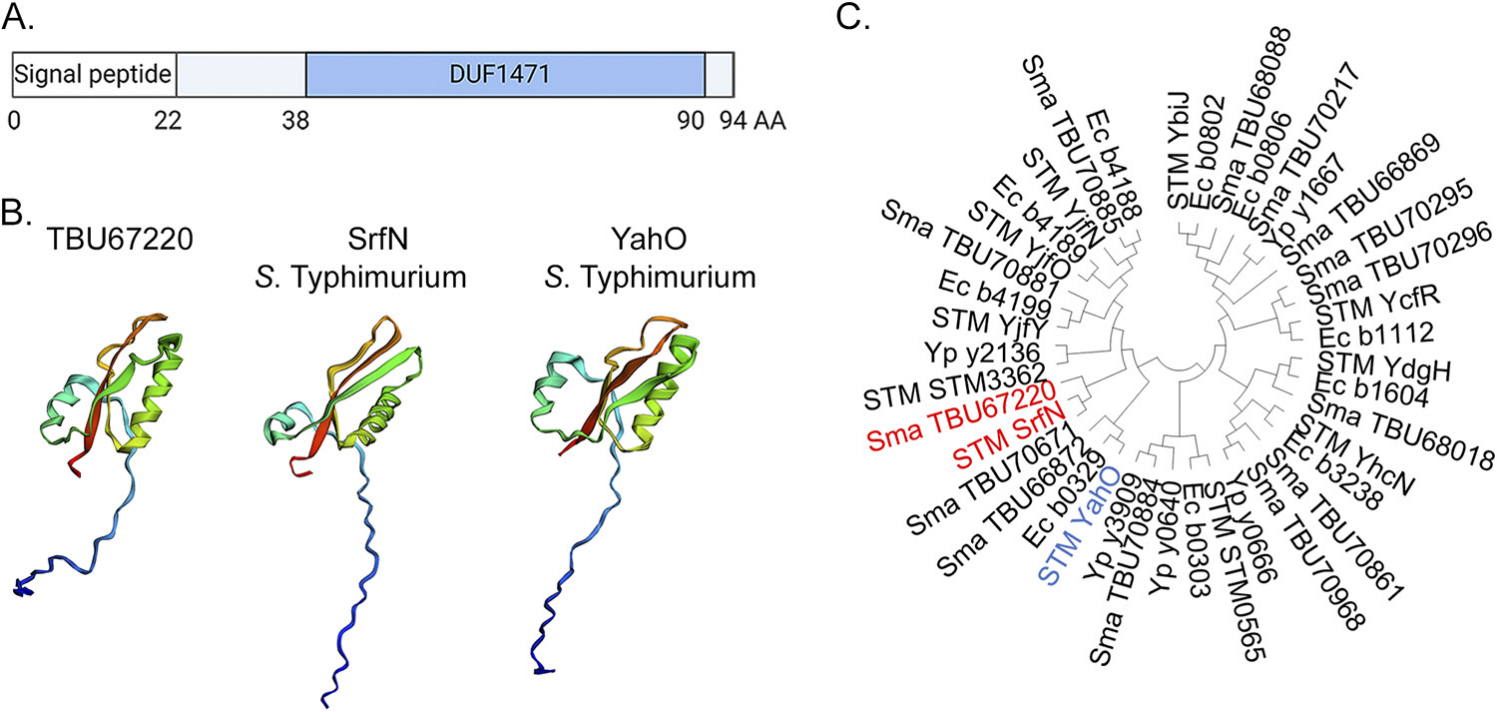
Low-molecular-weight secreted protein TBU67220 (SrfN) belongs to the DUF1471 superfamily. (A) SrfN domain architecture. Amino acid positions of the signal peptide and the DUF1471 domain are indicated. (B) SrfN structure prediction using the Phyre2 server, shown in comparison to the structures of Salmonella Typhimurium SrfN and YahO DUF1471-containing proteins. Protein structures are visualized using EzMol 2.1 (52). (C) The evolutionary history analysis of TBU67220 (SrfN) in comparison with other DUF1471-containing proteins from S. marcescens SM6 (Sma), Salmonella Typhimurium LT2 (STM), Escherichia coli K-12 (Ec), and Yersinia pestis KIM (Yp) was conducted in MEGA11 (28). The evolutionary distances were computed using the minimum evolution method (53) with the Poisson correction method and are shown in units of number of amino acid substitutions per site.

Since secreted S. marcescens metabolites with peroxide-protective properties are sensitive to heat and proteinase K treatment, we looked for the presence of extracellular proteins in the preconditioned medium fraction with a molecular weight of <10 kDa by using electrospray ionization–quadrupole time-of-flight mass spectrometry (ESI-QUAD-TOF/MS). Only two proteins were unambiguously identified in our sample, OsmY and a previously uncharacterized hypothetical protein (Table 1). Analysis of the amino acid sequence of the latter protein indicated that it corresponded to the Serratia marcescens SM6 TBU67220 protein. Evaluation of conserved domains within the TBU67220 amino acid sequence showed that this protein belongs to the family of DUF1471-containing proteins (Fig. 3A). Analysis of its amino acid sequence using the SignalP 5.0 prediction server (20) also suggested the presence of a signal peptide cleavage site between amino acid residues 22 and 23. Therefore, the mature TBU67220 protein is expected to consist of 72 amino acids. We prepared additional fractions of spent medium preconditioned by 4-h cultivation of wild-type bacteria in the presence or absence of peroxide (with molecular sizes of <3 kDa and >3 kDa) and analyzed them by multiple reaction monitoring (MRM) using a hybrid triple-quadrupole–linear ion trap (Q-TRAP) mass spectrometer. We found that TBU67220 was also present in the peroxide-exposed preconditioned medium fraction with a molecular weight of <3 kDa, likely due to partial leaking during centrifugal filtration, as shown for other proteins (21). Importantly, TBU67220 was not detected in the medium preconditioned by cultivation of wild-type S. marcescens in the absence of peroxide. Protein modeling using the Phyre2 server (22) showed that the TBU67220 protein fold closely resembles the three-dimensional model of the two previously structurally characterized DUF1471-containing proteins from Salmonella Typhimurium, SrfN and YahO (8) (Fig. 3B). The TBU67220 protein shared 47% identity and 68% similarity to *S.* Typhimurium SrfN and 42% identity and 62% similarity to the amino acid sequence of *S.* Typhimurium YahO proteins. Further evaluation of evolutionary relationship between all DUF1471-containing proteins present in Serratia marcescens SM6 (23), Salmonella Typhimurium LT2 (24), Escherichia coli K-12 (25, 26), and Yersinia pestis KIM (27) using MEGA11 (28) showed that TBU67220 appears to be more closely related to *S.* Typhimurium SrfN than to YahO (Fig. 3C). Therefore, we propose to name the previously uncharacterized low-molecular-weight DUF1471-containing S. marcescens SM6 protein TBU67220 SrfN, and we will use this name hereafter.

**TABLE 1:** Proteins identified in the low-molecular-weight fraction of the preconditioned medium using ESI-QUAD-TOF/MS

Protein name	MASCOT score	Swiss-Prot accession no.	*M*_r_ (Da)	SM6 protein ID	SM6 protein name	Protein size (aa)	Mature protein size (aa)
Hypothetical protein	996	BAC53662	10,111	TBU67220	DUF1471 domain-containing protein	94	72
Hypothetical protein	156	A0A1L6QL48	10,096				
Hypothetical protein	142	EZQ60896	10,110				
Putative periplasmic or secreted lipoprotein	186	AGB80957	21,826	TBU67655	Molecular chaperone OsmY	204	178
Periplasmic protein	128	AGE16453	21,147				

### The loss of *srfN* does not have an impact on the activity of extracellular enzymes.

**FIG 4:**
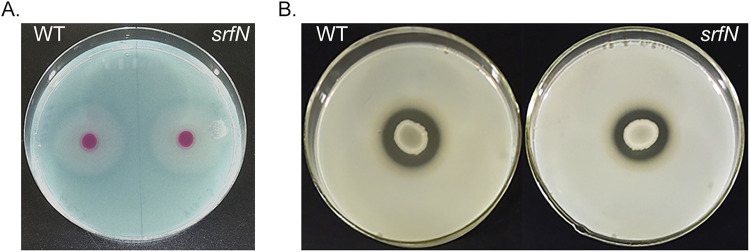
The loss of *srfN* does not impact activity of extracellular enzymes. (A) Nuclease activity of the wild type and the *ΔsrfN* mutant strains was tested on DNA-containing plates. Secretion of nuclease was detected by the formation of a halo around growing colonies. (B) Proteolytic activity of the wild type and the *ΔsrfN* mutant strains was tested on skim milk-containing agar plates. The clear zone around growing colonies corresponds to casein hydrolysis by secreted proteases. In both experiments, the overnight cultures of tested strains were normalized by the OD_600_ and used for spotting on the agar medium. Pictures were taken after incubation at 37°C for 24 h (A) or 48 h (B).

The available limited experimental data on DUF1471-containing proteins suggest that they may play a role in stress response and in surface colonization. S. marcescens is known to produce an array of extracellular enzymes (1, 29) attributed to the adaptation to new environments (2, 30). To test the involvement of the DUF1471-containing protein SrfN in the assortment of extracellular enzymes produced by this bacterium, we created a *ΔsrfN* mutant lacking the coding region of the gene and tested this strain for the production of an extracellular nuclease and proteases in a plate assay in comparison to the isogenic wild-type S. marcescens (Fig. 4). We found that both strains, the *ΔsrfN* mutant and the isogenic wild-type strain, were able to grow indistinguishably on plates containing DNA as the sole source of carbon and produced halos of comparable sizes. These data indicated that the growing colonies produced and secreted the active nuclease enzyme (Fig. 4A). Similarly, both strains secreted protease enzymes that were able to hydrolyze casein present in the skim milk agar plates, leading to the formation of a clear zone of a comparable size around mutant and wild type colonies (Fig. 4B). Thus, production of two classes of extracellular enzymes was not affected by elimination of the DUF1471-containing protein SrfN.

### SrfN is involved in S. marcescens adaptation to growth in low-pH medium.

**FIG 5:**
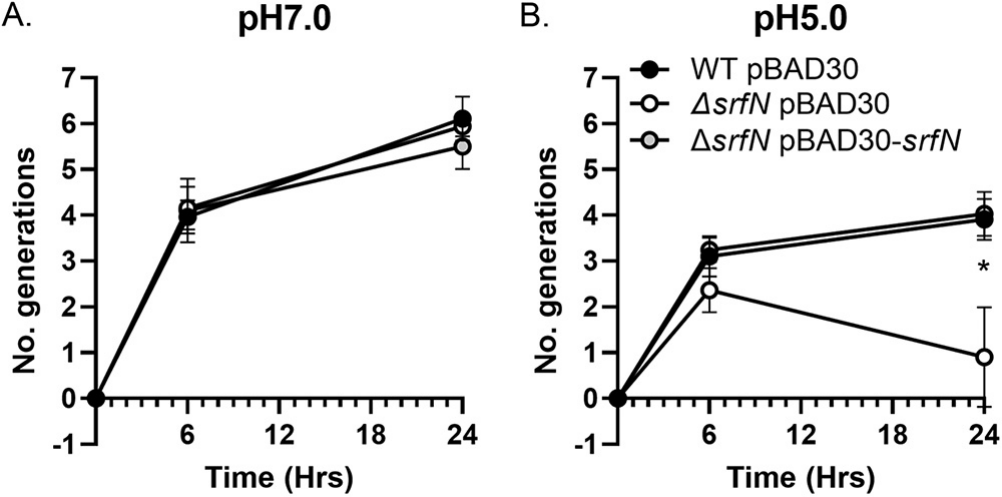
S. marcescens SrfN is involved in adaptation to growth in low-pH medium. Overnight LB-grown cultures of the wild type, the *ΔsrfN* mutant, and the *ΔsrfN psrfN* mutant strains were washed and subcultured at a 1:100 ratio in the MgM broth at pH 7.0 (A) or pH 5.0 (B). Aliquots were collected at the indicated time intervals, serially diluted, and plated. Data represent the number of generations from four independent experiments and the standard errors. *, *P* < 0.05 (unpaired *t* tests).

Since expression of some DUF1471-containing proteins is activated in response to a low-pH environment (31), we asked whether the mutant cells lacking *srfN* would be less fit in the low-pH medium compared to the wild type. In order to test our hypothesis, we grew the wild type, the *ΔsrfN* mutant, and the complemented *ΔsrfN* strain bearing a wild-type copy of the *srfN* gene on a plasmid, in a glycerol-containing MgM medium at neutral pH (pH 7) or low pH (pH 5). We found that all three strains were able to grow indistinguishably in the MgM medium at pH 7 (Fig. 5A). Furthermore, the wild-type *Serratia* strain was also able to multiply in the MgM medium at pH 5, albeit at a lower rate than in the same medium at neutral pH (Fig. 5B). In sharp contrast, while the *ΔsrfN* mutant was able to grow at pH 5 initially (after 6 h of incubation), the number of viable bacteria significantly declined after overnight growth. This growth defect was fully restored by complementation of the *ΔsrfN* mutant with a plasmid-borne *srfN* gene (Fig. 5B). We conclude that SrfN, a DUF1471-containing protein, is involved in S. marcescens adaptation to the acid stress.

### SrfN plays a role in S. marcescens protection from oxidative stress.

**FIG 6:**
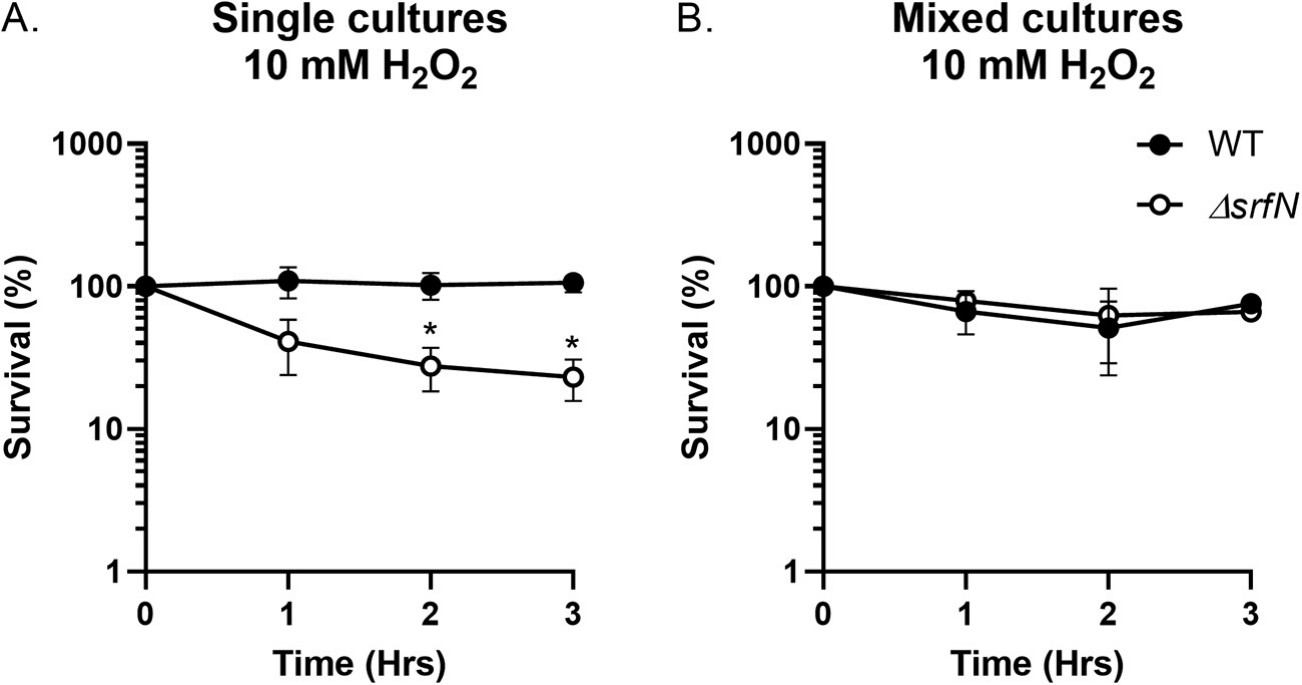
Wild-type-secreted SrfN restores growth of the *ΔsrfN* mutant in mixed S. marcescens cultures. (A) Overnight cultures of the wild type and the *ΔsrfN* mutant strains were subcultured in LB broth supplemented with 10 mM H_2_O_2_. (B) Individually grown overnight cultures of the wild type and the *ΔsrfN* mutant strains were mixed in an equal ratio, and the resulting mixture was subcultured in peroxide-containing LB broth. In both experiments, aliquots were collected hourly, serially diluted, and plated. Data represent the survival means from three independent experiments and the standard errors. *, *P* < 0.05 (unpaired *t* test).

Given that SrfN protein was identified in the minimal medium preconditioned by the growth of S. marcescens SM6 exposed to peroxide, we evaluated growth of the wild type and the *ΔsrfN* mutant in the medium containing H_2_O_2_. To ensure that the presence of the SrfN in the culture supernatant is not medium-dependent, we used rich LB broth supplemented with 10 mM hydrogen peroxide. As expected (19), the wild-type S. marcescens strain can tolerate this concentration of peroxide without changes in viability (Fig. 6A). The *ΔsrfN* mutant, however, was significantly more sensitive to H_2_O_2_ than the wild type. In contrast to the single cultures grown in peroxide, the presence of wild-type S. marcescens in the mixed culture experiment supported survival of the *ΔsrfN* mutant in 10 mM H_2_O_2_ (Fig. 6B).

**FIG 7:**
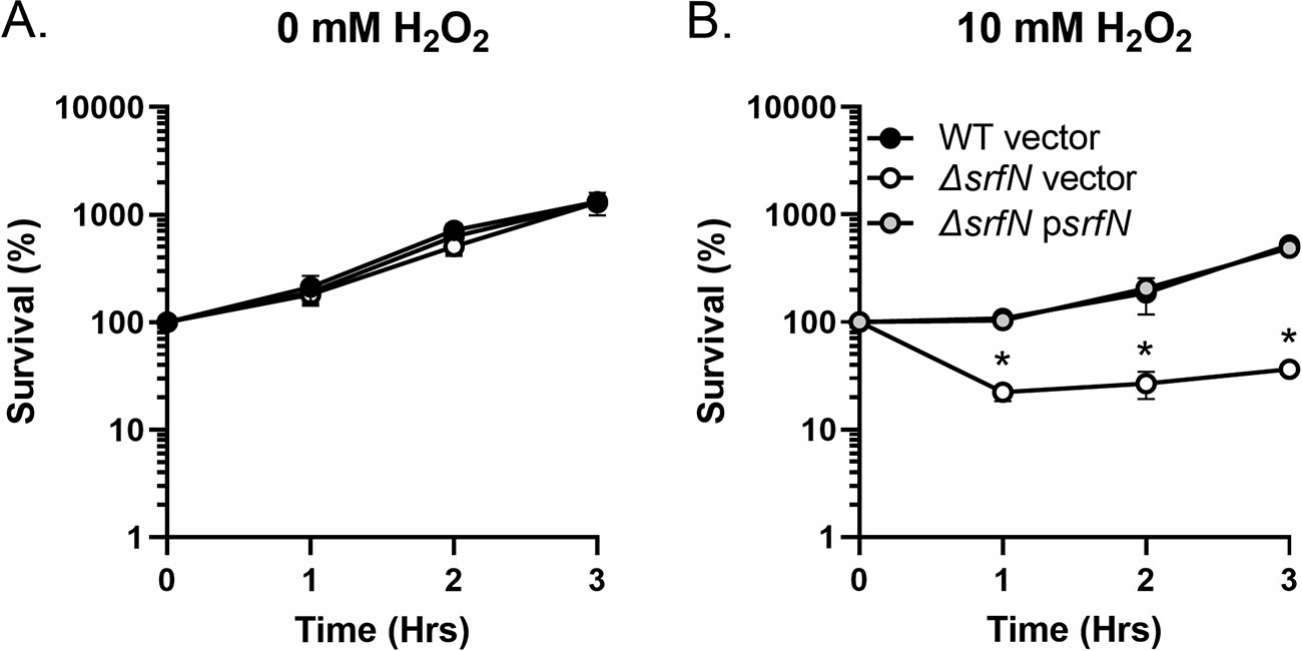
SrfN is involved in S. marcescens survival in the presence of peroxide. Overnight cultures of the wild type (black circles), the *ΔsrfN* mutant (white circles), and the *ΔsrfN* p*srfN* mutant (gray circles) strains were subcultured at a 1:100 ratio in LB broth containing no peroxide (A) or 10 mM H_2_O_2_ (B). Aliquots were collected hourly, serially diluted, and plated. Data represent the survival means from three independent experiments and the standard errors. *, *P* < 0.05 (unpaired *t* test).

Next, to directly assess the role of the DUF1471-containing SrfN protein in the ability of S. marcescens to withstand oxidative stress, we compared growth of the wild type, the *ΔsrfN* mutant, and the *ΔsrfN* strain bearing a plasmid-borne copy of the *srfN* gene in LB broth with or without 10 mM H_2_O_2_. All tested strains grew similarly in the absence of peroxide (Fig. 7A). However, exposure to H_2_O_2_ caused the *ΔsrfN* mutant bearing an empty vector to lose approximately 80% of its original population. Viability of the *ΔsrfN* mutant was fully restored by providing the intact copy of the *srfN* gene in *trans* (Fig. 7B).

**FIG 8:**
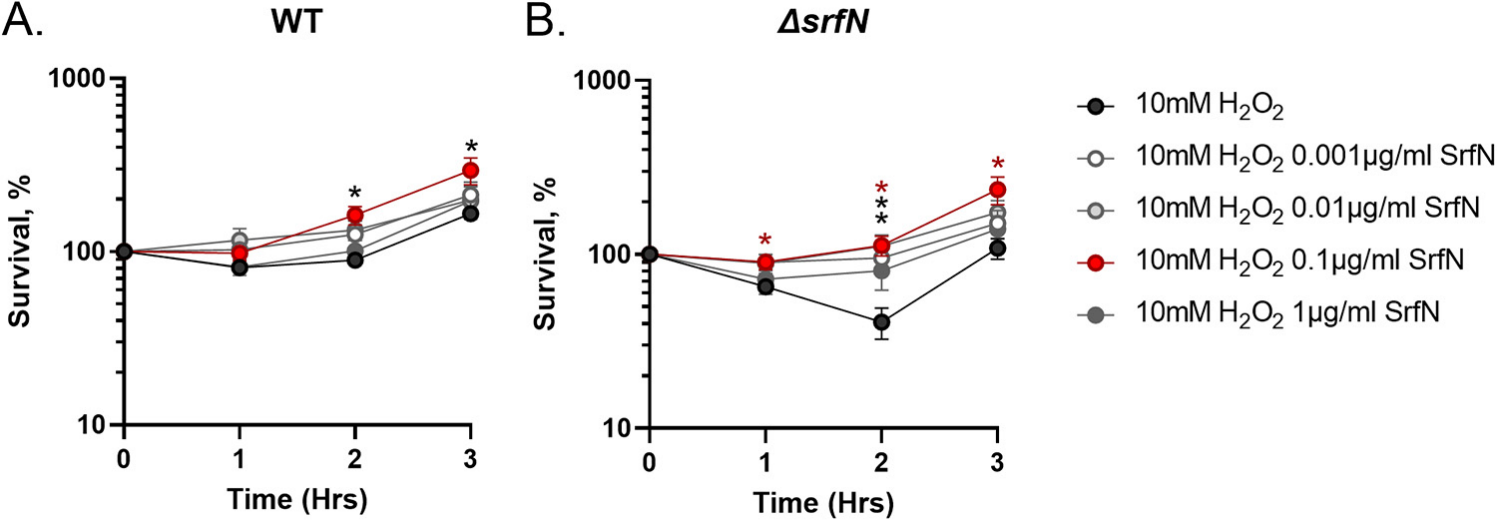
Exogenous SrfN protein is sufficient for S. marcescens *ΔsrfN* survival in the presence of peroxide. Overnight cultures of the wild type (A) and the *ΔsrfN* mutant strain (B) were subcultured at a 1:100 ratio in LB broth containing 10 mM H_2_O_2_ and 0 μg/mL (black circles), 0.001 μg/mL (white circles), 0.01 μg/mL (light gray circles), 0.1 μg/mL (red circles), or 1 μg/mL (gray circles) SrfN protein. Aliquots were collected hourly, serially diluted, and plated. Data represent the survival means from five independent experiments and the standard errors. *, *P* < 0.05 (unpaired *t* test).

Finally, we used chemically synthesized SrfN lacking signal peptide to address the question of whether peroxide protection is concentration dependent. We tested growth of the wild type and the *ΔsrfN* mutant in the medium containing H_2_O_2_ and increasing concentrations of the mature SrfN protein. We noticed that the addition of 0.1 μg/mL SrfN to the growth medium provided an additional protection from oxidative stress to the peroxide-resistant wild-type strain (Fig. 8A). Interestingly, exogenous SrfN also protected the *ΔsrfN* mutant from H_2_O_2_ damage in a dose-dependent manner. The addition of 0.001 or 0.01 μg/mL SrfN to the medium significantly improved *ΔsrfN* mutant survival at 2 h of growth in the presence of peroxide. Moreover, addition of 0.1 μg/mL SrfN fully protected the *ΔsrfN* mutant strain from the H_2_O_2_ toxicity. Interestingly, a further increase of exogenous SrfN to 1 μg/mL did not confer any significant benefits for *ΔsrfN* mutant strain survival in peroxide-containing medium (Fig. 8B), suggesting that the amount of available SrfN protein is important for its function.

Taken together, these data support the conclusion that the low-molecular-weight DUF1471-containing protein SrfN is involved in S. marcescens adaptation to oxidative stress.

## DISCUSSION

Bacteria employ several mechanisms to avoid and overcome oxidative stress. These mechanisms include a direct degradation of reactive oxygen species (ROS) by enzymes, including superoxide dismutases (SODs), catalases, and peroxidases. SODs convert superoxide anion into hydrogen peroxide. In turn, H_2_O_2_ is further degraded by catalases and peroxidases (16). In addition to the enzymatic ROS scavengers, catecholate siderophores and their derivatives have been reported to protect bacteria from oxidative stress (17, 32–34). The anti-H_2_O_2_ protection of Salmonella Typhimurium was recently proposed to be dependent on the secretion of a thermostable metabolite later identified as a linearized siderophore, enterobactin (17, 18). Here, we found that the supernatant of peroxide-exposed wild-type S. marcescens SM6 also harbored a metabolite(s) needed for growth of the extremely H_2_O_2_-sensitive mutant lacking a MacAB pump. Interestingly, the additional analysis of thermostability indicated that the antiperoxide protective effect of this S. marcescens*-*conditioned medium disappeared after boiling. This unexpected result highlighted the potential differences in anti-H_2_O_2_ protection mechanisms between distantly related bacterial species. Furthermore, the anti-H_2_O_2_ protective effect was also sensitive to proteinase K treatment, suggesting that the unknown metabolite(s) is likely a protein.

Liquid chromatography-tandem mass spectroscopy (LC-MS/MS) analysis of the low-molecular-weight (<10 kDa) fraction of the conditioned medium resulted in identification of only two polypeptides, OsmY and DUF1471-containing protein TBU67220. OsmY was originally identified in E. coli as a periplasmic protein induced in response to osmotic stress (35). It contains two BON domains (bacterial OsmY nodulation domains) and functions as a molecular chaperone involved in the biogenesis of autotransporters (36). Detection of OsmY in the S. marcescens extracellular milieu is not surprising, as the presence of this protein in the E. coli supernatant was reported previously (37). The second protein identified in our analysis, TBU67220, harbors a DUF1471 domain. The DUF1471 family consists of hundreds of proteins found only in bacteria from the order *Enterobacterales*; all of them are characterized by a small size (around 100 amino acids) and the presence of a signal peptide. Interestingly, a typical bacterium harbors multiple paralogs of DUF1471-containing proteins. For example, the genome of Salmonella Typhimurium contains 11 such genes, while the S. marcescens genome contains 14 genes coding for DUF1471-containing proteins (8, 23). However, our understanding of the role of the DUF1471-containing proteins in bacterial processes is very limited. To date, most studies have been related to a handful of *S.* Typhimurium proteins, namely, SrfN, YahO, and SssB (8). Those previous studies proposed that the function of these proteins could be related to the resilience of bacteria to environmental stress, to colonization of surfaces, or to their role in virulence. The Salmonella virulence program is controlled by a number of master regulators, including SPI-2 (Salmonella pathogenicity island 2) regulator SsrB (38). *S.* Typhimurium *srfN* was originally identified in a search for SsrB-dependent genes needed for bacterial fitness during systemic infection in mice (39) and was later shown to be translocated to the cytosol of infected macrophages by an unknown, type III secretion system-independent mechanism (40). More recently, *srfN* expression was also shown to be increased in a lettuce extract-containing medium (41). The role of DUF1471-containing proteins in the physiology of S. marcescens is currently unknown.

We have created a mutant strain lacking the production of TBU67220 (SrfN) and used it to identify phenotypes associated with the loss of this protein in S. marcescens. *Serratia* is known for a production of an array of extracellular enzymes with hydrolytic activities, including nuclease and proteases (2, 4, 5). The exact biological role of these enzymes is not completely understood, but it was hypothesized to be important for adaptation to a new niche (2, 30). Since the DUF1471-containing proteins can also be involved in colonization of new environments, we compared the production of nuclease and proteases by the *ΔsrfN* mutant and by the wild-type S. marcescens strain using plate assays. However, we could not detect any changes in the activities of extracellular enzymes between the two strains.

Expression of the *S.* Typhimurium *yahO* gene encoding a DUF1471-containing protein was previously found to be upregulated in poor medium with low pH (31). The bacterial acid stress response is focused on the prevention of a drop in intracellular pH. This could be achieved via three major mechanisms: through enzymatic reactions that consume protons, such as decarboxylation of amino acids; through production of pH-neutralizing compounds (production of ammonia from urea); and finally, through proton removal by F_1_F_0_-ATPase. In addition to these main mechanisms, bacteria can protect themselves from acid stress through modification of the lipid composition of the cytoplasmic membrane or through protein refolding (42). The exact role of DUF1471-containing proteins in bacterial adaptation to low pH is yet to be defined, but our data indicate that the S. marcescens *ΔsrfN* mutant was less fit in the minimal MgM medium at pH 5, and this growth defect was rescued by complementation.

Finally, the SrfN DUF1471-containing protein identified in our study was present in the extracellular milieu of S. marcescens exposed to peroxide. Several other DUF1471-containing proteins have also been reported to be associated with bacterial resistance to oxidative stress (9, 11–13). Our experiments confirmed that the mutant lacking SrfN was more sensitive to peroxide than the wild-type strain, and this phenotype was reverted by coculture with wild-type S. marcescens, by complementation in *trans*, and by addition of the chemically synthesized protein to the growth medium.

In conclusion, our study identified a new, previously uncharacterized DUF1471-containing protein in Serratia marcescens that is needed for adaptation of this bacterium to acid and oxidation stress.

## MATERIALS AND METHODS

### Bacterial strains, media, and growth.

All S. marcescens strains used in this study are listed in Table 2. Strains were grown in LB broth (10 g tryptone, 5 g yeast extract, 5 g NaCl per liter) or a minimal M9 medium (6.77 g Na_2_HPO_4_, 3 g KH_2_PO_4_, 1 g NH_4_Cl, 0.5 g NaCl, 2% glycerol per liter; pH 7.0). When needed, antibiotics were used at the following concentrations: 50 mg/liter kanamycin, 100 mg/liter carbenicillin. Bacteria were grown at 30°C with shaking (250 rpm) unless stated otherwise.

**TABLE 2:** Strain list

Strain	Description	Reference
LMB1	S. marcescens SM6 wild type	23
LMB71	S. marcescens SM6 *ΔmacAB*::Cm^R^	19
LMB457	S. marcescens *SM6 ΔsrfN*::Kan	This study
LMB430	S. marcescens SM6 pBAD30, Amp^R^	19
LMB458	S. marcescens *SM6 ΔsrfN*::Kan pBAD30, Amp^R^	This study
LMB459	S. marcescens *SM6 ΔsrfN*::Kan pBAD30-*srfN*, Amp^R^	This study

### Sensitivity of S. marcescens strains to hydrogen peroxide.

Overnight cultures of the wild type or the *ΔmacAB* mutant strain (19) were grown in LB, washed twice with phosphate buffer (pH 7.0), and subcultured at a 1:100 ratio in M9 glycerol-containing medium with 0, 3, 4, 5, or 6 mM H_2_O_2_ (AppliChem). For complementation studies, cultures of the wild type carrying an empty pBAD30 plasmid, the *ΔsrfN* pBAD30 strain, and the *ΔsrfN* p*srfN* mutant strains were grown overnight in LB and subcultured at a 1:100 ratio in fresh LB broth containing either no peroxide or 10 mM H_2_O_2_ (AppliChem). The resulting cultures were incubated at 37°C with shaking. Aliquots were collected hourly, serially diluted, and plated for CFU enumeration. Results are expressed as the percent survival [CFU(*t_n_*)/CFU(*t*_0_)] × 100 over time; CFU(*t_n_*) is the CFU recovered at specific time point n, and CFU(*t*_0_) is the starting CFU inoculum at time zero. Experiments were done on at least three separate occasions.

### Preparation of Serratia marcescens SM6-conditioned medium.

Overnight culture of the wild-type strain was grown in LB, washed twice with phosphate buffer (pH 7.0), subcultured in M9 glycerol-containing medium at a 1:100 ratio containing 6 mM H_2_O_2_ (AppliChem), and incubated at 37°C for 4 h with shaking. Conditioned medium was separated from cells by 20 min of centrifugation at 10,000 rpm, 4°C (Hermle) followed by filtration through 0.2-μm-pore-size filters (Corning) to remove any remaining bacteria. The resulting metabolite-containing medium was supplemented with 6 mM H_2_O_2_ and used for growth of a peroxide-sensitive *ΔmacAB* indicator strain prepared as described above. Additional tubes containing M9 medium with no peroxide or with 6 mM H_2_O_2_ were inoculated with the *ΔmacAB* mutant strain as positive and negative controls for bacterial growth, respectively. Bacterial cultures were incubated at 37°C with shaking. Aliquots were collected hourly, serially diluted, and plated for CFU enumeration. Results are expressed as the percent survival [CFU(*t_n_*)/CFU(*t*_0_)] × 100 over time. Experiments were done on at least three separate occasions.

### Heat and proteinase K treatment.

S. marcescens SM6-conditioned medium was prepared as described above. The resulting preconditioned medium was divided into three tubes. One tube’s contents were boiled for 10 min and cooled on ice. The content of the other tube was incubated with 50 μg/mL proteinase K (ThermoFisher) for 3 h at 55°C. The remaining tube was left untreated. After treatment, 6 mM peroxide was added to each tube and used to determine growth of the *ΔmacAB* mutant strain. Bacterial cultures were incubated at 37°C with shaking. Aliquots were collected hourly, serially diluted, and plated for CFU enumeration. Results are expressed as the percent survival [CFU(*t_n_*)/CFU (*t*_0_)] × 100 over time. Experiments were done on at least three separate occasions.

### Fractionation of S. marcescens SM6-conditioned medium.

Overnight culture of the wild-type strain was grown in LB, washed twice with a phosphate buffer (pH 7.0), and subcultured in 1 liter of M9 medium containing 6 mM H_2_O_2_ at a 1:100 ratio. After 4 h of incubation at 37°C with shaking, preconditioned medium was prepared as described above. Filtered metabolite-containing medium was further stepwise separated using Amicon Ultra centrifugal filters with molecular weight cutoffs of 3, 10, and 30 kDa. The resulting fractions containing metabolites with a molecular weight of <3 kDa (fraction 1), 3 to 10 kDa (fraction 2), 10 to 30 kDa (fraction 3), and >30 kDa (fraction 4) were supplemented with 6 mM H_2_O_2_ and used for growth of a peroxide-sensitive *ΔmacAB* indicator strain prepared as described above. Bacterial cultures were incubated at 37°C with shaking. Aliquots were collected hourly, serially diluted, and plated for CFU enumeration. Results are expressed as the percent survival [CFU(*t_n_*)/CFU (*t*_0_)] × 100 over time. Experiments were done on at least three separate occasions.

### Identification of low-molecular-weight proteins in S. marcescens SM6-conditioned medium.

Metabolite-containing medium was prepared as described above. Low-molecular-weight proteins (<10 kDa) were collected using an Amicon Ultra centrifugal filter with a molecular weight cutoff of 10 kDa. The protein concentrations were measured by the Lowry method with bicinchoninic acid (43). One hundred micrograms of protein was used for separation on the polyacrylamide gel and stained with Coomassie blue G-250. The protein-containing band was excised from the gel and separated into 1- by 1-mm sections. The dye was removed from the samples by washing them twice with 100 mM ammonium bicarbonate–50% acetonitrile solution (vol/vol) at 50°C for at least 30 min until the color disappeared. Gel fragments were dehydrated by a 20-min incubation in 100% acetonitrile followed by overnight trypsin treatment (1 mg enzyme/50 mg protein) at 37°C. The reaction was stopped with 0.1% trichloroacetic acid (TCA), and the peptides were recovered from the gel by sonication in an ultrasonic bath (S30 Elmasonic, Elma). Peptide solution was transferred to a clean tube and extracted from the gel with 70% acetonitrile in water. Peptide-containing supernatant was collected and dried on a centrifugal evaporator (Eppendorf) at 45°C. Samples were reconstituted in 100 μL of solvent A (5% acetonitrile–0.1% formic acid [FA] in water) and used for the LC-MS/MS. The resulting peptides were separated on an AcclaimPepMap C_18_ column (2-μm pore size; 100 Å; 75 μm by 15 cm [Thermo Scientific]) and analyzed on a Dionex Ultimate 3000 system (Thermo Scientific) coupled with a maXis impact mass spectrometer (Bruker) as described previously (44), with modifications. The mobile phase consisted of 5% acetonitrile–0.1% FA in water (solvent A) and 94.9% acetonitrile–0.1% FA in water (solvent B). Chromatographic separation was performed in the following steps: 0 to 5 min, 2% solvent B; 5 to 60 min, 2 to 45% gradient solvent B; 60 to 61 min, 90% solvent B; 71 to 75 min, 90 to 2% gradient solvent B; 75 to 80 min, 2% solvent B, with a flow rate of 300 nL/min at 40°C. A spectrum of positively charged ions was collected using a 3-liter/min gas flow rate, 1,600 V at 150°C with a 50 to 2,200 *m/z* detection range, 10-Hz sampling frequency, and automatic ion fragmentation in MS/MS mode.

Obtained mass spectra were processed using DataAnalysis 4.1. Proteins were identified using Mascot 2.4.0 against the Serratia marcescens NCBI database, based on the detection of at least two unique peptides with a score >35.

### TBU67220 (SrfN) detection in S. marcescens SM6-conditioned medium.

Metabolite-containing media samples used for growth of the wild-type strain in the presence or in absence of 6 mM H_2_O_2_ were prepared as described above. Low-molecular-weight proteins (<3 kDa) were separated from larger proteins by using an Amicon Ultra centrifugal filter with a molecular weight cutoff of 3 kDa. The protein concentration in each sample was determined as described above. Proteins were digested with ProteaseMAX surfactant (Promega) according to the manufacturer’s recommendations. The resulting peptides were separated on the Titan C_18_ column (1.9 μm by 10 cm by 2.1 mm; Supelco) and analyzed on an Infinity 1290 system (Agilent) coupled with a Q-TRAP 6500 mass spectrometer (AB Sciex). The mobile phase consisted of 5% acetonitrile–0.1% FA in water (solvent A) and 94.9% acetonitrile–0.1% FA in water (solvent B). Chromatographic separation was performed in the following steps: 0 to 17 min, 3 to 40% gradient solvent B; 17 to 18 min, 40 to 95% gradient solvent B; 18 to 21.5 min, 95% solvent B; 21.5 to 23 min, 95 to 3% gradient solvent B; 23 to 25 min, 3% solvent B, with a flow rate of 0.4 mL/min at 40°C.

The Turbo V IonDrive ion spray source was set at 5,200 V, and 500°C was used for electrospray ionization. The instrument was operated in positive ion mode with a curtain gas set at 35 lb/in^2^, nebulizer gas set at 60 lb/in^2^, and auxiliary gas of the mass spectrometer set at 60 lb/in^2^. The collision energy (CE) and declustering potential (DP) for TBU67220 (SrfN)-specific peptides were determined using Skyline 21.2 software. TBU67220-specific peptides GGQYYVILAGR and FSAIAEVYK were monitored by MRM with mass transitions from *m/z* 598.824 to *m/z* 668.303/791.477/628.414 at CE of 30.4 V and DP of 74.8V, and *m/z* 514.276 to *m/z* 619.308/718.377/881.440 at CE of 27.4 V and DP of 68.6. Collected chromatograms were analyzed using MultiQuant 3.0.2 software.

### Generation of S. marcescens *ΔsrfN* deletion mutant.

The *ΔsrfN* mutant was generated by lambda red recombinase-mediated homologous recombination (45–47) using primers srfN-KO-Fwd (GGTTAGTGTCATGAAATCATTGAAGATGATTATTGCAGCGGCAGTGCTGGGGCTGGAGCTGCTT), srfN-KO-Rev (CGTTCAAGGCTTATTGTTTGTCTTTGTAAACTTCGGCGATGGCGCTGAATGAATATCCTCCTTAG), and a template plasmid, pCLF4 (45). The resulting mutant clones were confirmed by PCR.

### Plasmid construction.

A complementing plasmid carrying the *srfN* gene was generated as follows. A DNA fragment containing the full-length open reading frame with 500 bp upstream of *srfN* was amplified by PCR using genomic DNA of S. marcescens SM6 and primers XbaI-srfN-Fwd (5′-ACTCTAGAAGATCCTGCGCACCGCCG-3′) and HindIII-srfN-Rev (5′-ATAAGCTTTTAGTGGTGATGGTGATGATGTTGTTTGTCTTTGTAAACTTC-3′), respectively. PCR product was digested with XbaI and HindIII and used for ligation into vector pBAD30 (48) previously treated with the same enzymes. Clones with the correct insertion were confirmed by restriction digestion and sequencing.

### Plate assay for extracellular enzyme activities.

To test the ability of the Δ*srfN* mutant strain to secrete an active nuclease, overnight cultures of the wild type and the *ΔsrfN* mutant strain were grown in LB broth, normalized to the optical density at 600 nm (OD_600_), and washed once with 0.9% NaCl solution. Five microliters of each strain was spotted on agar plates containing 300 mg bovine spleen DNA (Reachim, Russia), 1% agar, 0.1% 0.01 M CaCl_2_, 1% NaCl, and 0.3% 0.1 M toluidine blue (49). The nuclease secretion led to a color change around the bacterial colonies. The zone diameters were measured after 24 h of incubation at 37°C.

The ability of the Δ*srfN* mutant strain to secrete an active protease was tested on skim milk agar (1% tryptone, 0.5% yeast extract, 1% NaCl, 3% skim milk, 2% agar) (50). Overnight cultures of the wild type and the Δ*srfN* mutant strain were prepared as described above, and 5 μL of each strain was spotted on skim milk agar plates and incubated for 48 h at 37°C. The production of proteases led to the formation of a transparent zone around the growing bacterial colony. The diameter of this zone was measured after 24 and 48 h of incubation. Experiments were done in triplicate.

### S. marcescens growth in medium with low pH.

Overnight cultures of wild type carrying an empty pBAD30 plasmid and *ΔsrfN* pBAD30 and *ΔsrfN* p*srfN* mutant strains were grown in LB broth, washed twice with a minimal MgM medium [100 mM Tris-HCl (pH 7), 5 mM KCl, 7.5 mM (NH_4_)_2_SO_4_, 0.5 mM K_2_SO_4_, 1 mM KH_2_PO_4_, 8 μM MgCl_2_, 2% glycerol, and 0.1% Casamino Acids] (51) and subcultured at a 1:100 ratio in fresh MgM medium, pH 5. In parallel, the same bacterial cultures were inoculated at a 1:100 ratio in the MgM medium, pH 7. Aliquots were collected at 0, 6, and 24 h of growth at 37°C, serially diluted, and plated on LB agar for CFU enumeration. Data were expressed as the number of generations calculated using the following equation: [log_10_(CFU_final_) − log_10_(CFU_start_)]/log_10_(2). The experiment was done in four replicates.

### Hydrogen peroxide sensitivity of mixed cultures in cross-complementation studies.

Overnight cultures of wild type and *ΔsrfN* mutant strains were grown in LB broth and combined at a 1:1 ratio. The resulting mixed culture was diluted 1:100 in fresh LB broth supplemented with 10 mM H_2_O_2_ and incubated at 37°C with shaking. Aliquots were collected hourly, serially diluted, and plated on LB agar supplemented with appropriate antibiotics for CFU enumeration. Results were expressed as percent survival [CFU(*t_n_*)/CFU (*t*_0_)] × 100 over time. Experiments were done on at least three separate occasions.

### Hydrogen peroxide sensitivity of the *ΔsrfN* mutant in the presence of exogenous SrfN protein.

The mature version of the SrfN protein lacking signal peptide was chemically synthesized by Biomatik Corporation. Overnight cultures of the wild-type and the *ΔsrfN* mutant strains were grown in LB broth and diluted 1:100 in fresh LB broth supplemented with 10 mM H_2_O_2_ and 0, 0.001, 0.01, 0.1, or 1 μg/mL SrfN protein. The resulting cultures were incubated at 37°C with shaking. Aliquots were collected hourly, serially diluted, and plated for CFU enumeration. Results were expressed as percent survival [CFU(*t_n_*)/CFU(*t*_0_)] × 100 over time. Experiments were done on at least five separate occasions.

### Data analysis.

Statistical significance (*P* < 0.05) was determined using the unpaired *t* test with Welch correlation. Analyses were performed using GraphPad Prism v.9.2.0.
